# International Hahnemannian Association

**Published:** 1894-11

**Authors:** 


### INTERNATIONAL HAHNEMANNIAN ASSOCIATION.

The President takes pleasure in reminding those members who were present, and informing those who were absent, deterred by professional duty or possibly by the love of travel, an unusual opportunity to explore the mountains of Colorado, the social institutions of Utah, or the geysers of the Yellowstone Park, of the highly interesting character of the proceedings of the International Hahnemannian Association in June last at Niagara. The Association sustained its prestige in all its Bureaux by the presentation of papers, philosophical and practical, clinical reports, discussions, and illustrative experiences not hitherto excelled.

As the conservator and practical teacher of pure Homœopathy, its friends and members will maintain its honor and extend its influence and increase its membership. Those who may not yet be fully qualified for membership have as associate members the opportunity to witness the verification of the therapeutic science taught by Hahnemann.

Application blanks for membership and associate membership may be obtained of the Chairman of the Board of Censors, Dr. A. R. Morgan, Waterbury, Conn.

